# Performance of stool-based Xpert MTB/RIF assay in the diagnosis of presumptive pulmonary tuberculosis in adults unable to expectorate sputum

**DOI:** 10.1128/spectrum.02495-24

**Published:** 2025-02-25

**Authors:** Yi Xue, Siyi Chen, Weihe Zhang, Junnan Jia, Fen Wang, Lingling Dong, Liping Zhao, Hairong Huang, Xia Yu

**Affiliations:** 1 National Clinical Laboratory on Tuberculosis, Beijing Key Laboratory for Drug Resistant Tuberculosis Research, Beijing Chest Hospital, Capital Medical University, Beijing Tuberculosis and Thoracic Tumor Institute, Beijing, China; Icahn School of Medicine at Mount Sinai, New York, New York, USA

**Keywords:** pulmonary tuberculosis, Xpert MTB/RIF, stool, adult

## Abstract

This study aimed to compare the performance of stool-based Xpert MTB/RIF(Xpert) assay (Cepheid, USA) against that of smear, culture, and Xpert using respiratory tract specimens (RTS) and stool samples in the diagnosis of adult pulmonary tuberculosis (PTB). This prospective study of patients with presumptive PTB was conducted at the Beijing Chest Hospital from January 2021 to December 2021. The smear test by fluorescence microscope, MGIT 960 liquid culture, and Xpert MTB/RIF assay was performed simultaneously on the RTS and stool samples. Patients were grouped based on the RTS examination results and clinical diagnosis. A total of 434 eligible patients were enrolled. Among these, 351 patients were diagnosed as PTB, while 83 patients were diagnosed as non-TB patients. Using RTS as standard sample, the sensitivity of smear on stool, culture on stool, Xpert on stool, and Xpert on RTS was 13.39% (47/351), 22.22% (78/351), 45.30% (159/351), and 66.10% (232/351), respectively. Furthermore, the specificity of Xpert on RTS and stool was 100% (83/83) for both specimens. Among the 48 patients with PTB confirmed by bronchoalveolar lavage fluid (BALF) examination, 16 had stool specimens with positive Xpert results. Similarly, among 200 patients diagnosed through sputum examination, 133 had positive Xpert results on stool specimens. Thus, Xpert on stool specimens may be a promising and practical strategy to improve PTB diagnosis, particularly among patients unable to expectorate sputum.

**IMPORTANCE:**

This study aimed to assess the value of the Xpert MTB/RIF assay (Xpert) for diagnosing PTB using stool samples from adults in low HIV prevalence settings. Although the diagnostic yield of Xpert on stool samples is inferior to that of Xpert on RTS, stool-based Xpert may be useful in diagnosing patients with presumptive PTB who cannot expectorate sputum and do not opt for BALF collection.

## INTRODUCTION

The World Health Organization (WHO) estimates that 10.6 million people were diagnosed with tuberculosis (TB) and 1.3 million TB-related deaths had occurred worldwide in 2022 (1). The missed or delayed diagnosis of TB is the primary cause of morbidity from this disease. Conversely, the early detection of TB lowers transmission risk and improves the effectiveness of anti-TB treatment. However, the current diagnostic methods (such as traditional smear microscopy and culture tests) and molecular diagnostic techniques remain insufficient to achieve the goals of the WHO End TB Strategy.

Pulmonary tuberculosis (PTB) in adults accounts for 90% of the reported PTB cases (2). Currently, the bacteriological diagnosis of PTB in adults mainly relies on sputum examination with smear, culture, and molecular assays, including the Xpert MTB/RIF assay (Xpert) (3). Molecular diagnostic tests have been developed to reduce the diagnostic gaps and delays in initiating treatment. Xpert on sputum specimens is widely considered the initial diagnostic test for PTB. Using sputum culture as the gold standard, the sensitivity and specificity of Xpert using sputum samples are 84.7% (95% CI: 78.6%–89.9%) and 98.4% (95% CI: 97.0%–99.3%), respectively (4). Simultaneously, the performance of these assays is highly dependent on the quality and volume of the sputum specimen. In addition, previous studies showed the sensitivity of Xpert assay on urine were 33% and 57%, and the specificity of Xpert on urine were both 100% (5, 6). For blood sample , Xpert assay owned a high specificity of 99%–100% and low sensitivity of 7.1%–21% (7, 8). Adults with minimally symptomatic TB and those admitted to the hospital are often unable to provide an adequate sputum sample (9). In such situations, induced sputum and bronchoalveolar lavage fluid (BALF) serve as the alternative specimen types for diagnostic analysis. Sputum induction utilizing an ultrasonic nebulizer is completed in approximately 20 min each time and must be performed at least twice, making it an inconvenient procedure (10). Although previous studies have shown that BALF obtained good yields for molecular testing (49%–63%) in patients with smear-negative PTB, the invasive and expensive features of this bronchoscopy technique limit its widespread usage. Therefore, novel diagnostic strategies using feasible alternatives to sputum are urgently required.

Patients with TB may swallow sputum when they have difficulty coughing it up, carrying MTB into the gastrointestinal tract. In addition, MTB is transmitted through the blood or lymphatic system and may colonize in the gastrointestinal tract and be transferred to the stool via gastrointestinal-associated lymphoid tissue (11). Respiratory secretions are routinely emptied into the gastrointestinal tract. Stool is one of the gastrointestinal tract specimens that can be easily obtained without any invasive procedure (12–16). Several studies have assessed the performance of Xpert on stool specimens from adults (17–19). For instance, our previous studies have indicated that the sensitivity of Xpert on stool was 45.83% (95% CI: 35.73–56.28), while 13.8% (4/29) of patients with possible PTB were categorized with bacteriologically confirmed TB using stool-based Xpert results (19). Rahman et al. also demonstrated that Xpert on stool specimens could identify 94.8% (95% CI: 88.5–97.8) of adults with sputum culture-positive PTB (18). Furthermore, a meta-analysis of three studies evaluated the performance of Xpert on stool specimens in diagnosing TB in adults, and the results revealed that the sensitivity and specificity of Xpert were 85.7%–90.6% and 93.9%–100%, respectively (18, 20, 21). Although stool-based Xpert is not yet recommended in adult guidelines for TB diagnosis, stool testing represents a new, crucial diagnostic strategy for adult PTB, especially in patients unable to expectorate sputum. In addition, according to the latest report from the Chinese center for disease control and prevention, the prevalence of HIV infection in China for adults is approximately 0.1%–0.2%, which is in the global low-prevalence range (22). Therefore, this study compared the performance of stool-based Xpert with that of various tests using stool samples and respiratory tract specimens (RTS) from adults with presumptive PTB in low HIV settings.

## RESULTS

### Study population

**Fig 1:**
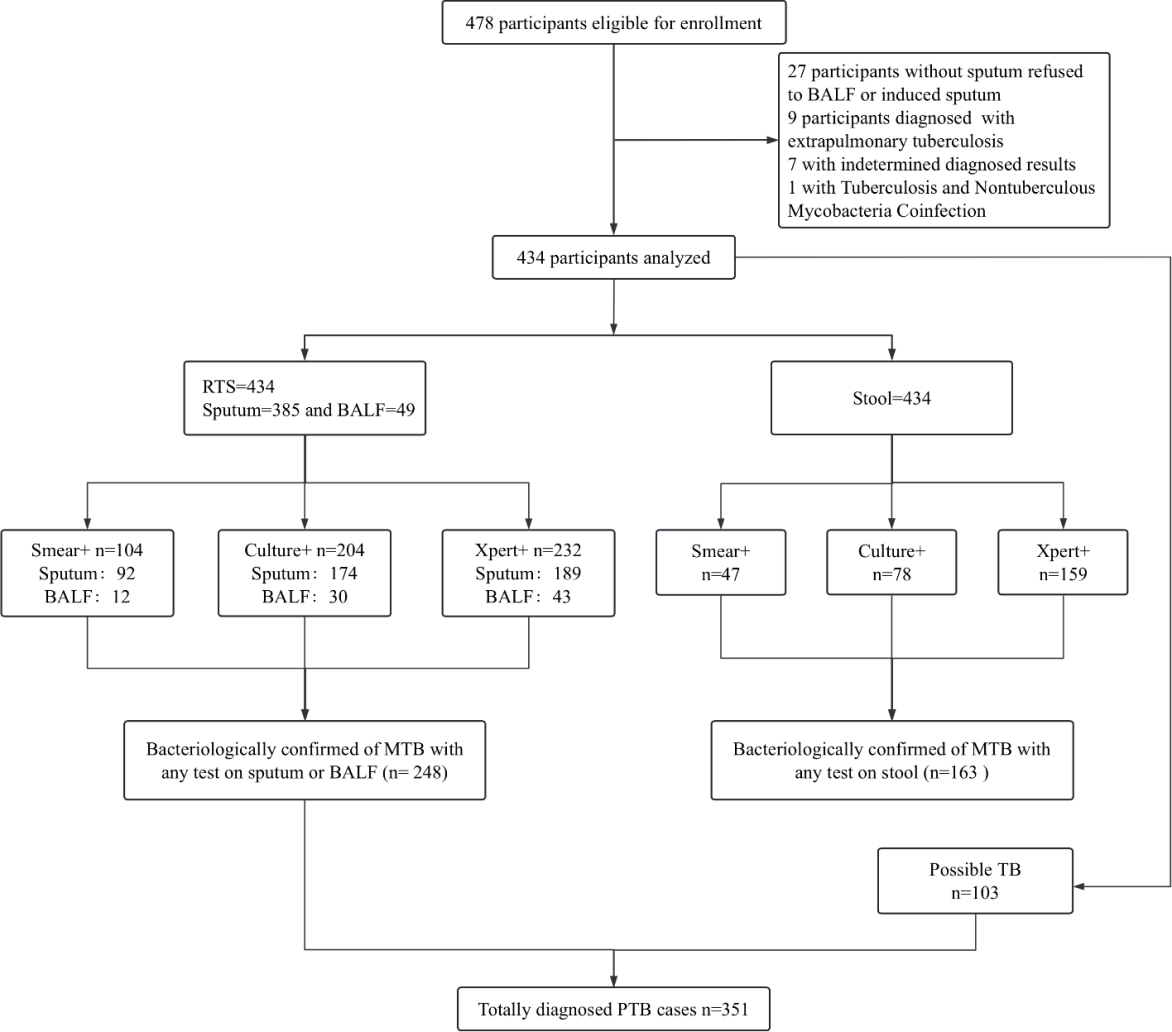
Flowchart depicting the enrollment, and investigation results with presumptive pulmonary tuberculosis in adults. BALF, bronchoalveolar lavage fluid; RTS, respiratory tract specimens.

A total of 478 adult patients with presumptive PTB were enrolled, from which 44 were excluded. Among the 44 excluded patients, 27 were unable to expectorate sputum and refused to undergo bronchoscope examination, 9 were diagnosed with extrapulmonary TB, 7 had an undetermined diagnosis, and 1 had co-infection with tuberculous and nontuberculous mycobacteria (NTM). Ultimately, specimens from the remaining 434 patients were used to perform all the required tests in this study. According to the tests on RTS, 248 patients were diagnosed with confirmed TB, whereas 103 were diagnosed with probable TB. In the bacteriologically confirmed TB group, the positive detection rates of smear, culture, and Xpert for respiratory tract specimens (RTS) were 41.13% (102/248), 76.61% (190/248), and 93.55% (232/248), respectively. The corresponding positive detection rates for stool specimens were 17.34% (43/248), 28.23% (70/248), and 60.08% (149/248), respectively. In the probable TB group, the positive detection rates of smear, culture, and Xpert on stool were 1.94% (2/103), 3.88% (4/103), and 9.71% (10/103), respectively. The remaining 83 patients were classified as non-TB, including 12 with lung cancer diagnosed by tissue pathology and 71 with other respiratory infectious diseases. Among these, five patients yielded positive cultures, which were identified as *Mycobacterium intracellulare* by *16S rRNA*, *hsp65*, *rpoB*, and *16S-23S rRNA* internal transcribed spacer genes sequencing, were grouped into the non-TB category (Fig. 1). Furthermore, all patients were HIV-negative. The demographics and clinical characteristics of all enrolled patients are presented in Table 1, and there is no significant differences in demographics, TB history, and history of TB contact among the three patient groups.

**TABLE 1:** Demographic and clinical characteristics of the study participants

	TB (*n* = 351)				
Characteristics	Total (*n* = 351)	Confirmed TB (*n* = 248)	Probably TB (*n* = 103)	Non-TB (*n* = 83)	*P* value
Gender:male/female					0.840
Male	237 (67.5%)	173 (69.8%)	64 (62.1%)	57 (68.7%)	
Female	114 (32.5%)	75 (30.2%)	39 (37.9%)	26 (31.3%)	
Age, average (range)	54 (18–88)	53 (18–88)	55 (18–87)	56 (22–83)	0.328
TB history					0.494
Yes	102 (29.1%)	73 (29.4%)	29 (28.2%)	21 (25.3%)	
No	249 (70.9%)	175 (70.6%)	74 (71.8%)	62 (74.7%)	
On tuberculosis treatment					0.205
Yes	158 (45.0%)	99 (39.9%)	59 (57.3%)	31 (37.3%)	
No	193 (55.0%)	149 (60.1%)	44 (42.7%)	52 (62.7%)	
History of TB contact					0.213
Yes	26 (7.4%)	22 (8.9%)	4 (3.9%)	3 (3.6%)	
No	325 (92.6%)	226 (91.1%)	99 (96.1%)	80 (96.4%)	
Weight loss					0.172
Yes	104 (29.6%)	80 (32.3%)	24 (23.3%)	31 (37.3%)	
No	247 (70.4%)	168 (67.7%)	79 (76.7%)	52 (62.7%)	
Cough >2 week					0.438
Yes	272 (77.5%)	210 (84.7%)	62 (60.2%)	61 (73.5%)	
No	79 (22.5%)	38 (15.3%)	41 (39.8%)	22 (26.5%)	
Fever					0.272
Yes	137 (39.0%)	107 (43.1%)	30 (29.1%)	27 (32.5%)	
No	214 (61.0%)	141 (56.9%)	73 (70.9%)	56 (67.5%)	

### Performance of different tests in PTB diagnosis

During RTS collection from the 434 patients, sputum samples were obtained from 385, and BALF specimens were collected from 49. In the sputum tests, 92 patients were positive by smear, 174 by culture, and 189 by Xpert. In the case of the BALF tests, 12 were positive by fluorescent smear microscopy, 30 by liquid culture (MGIT960), and 43 by Xpert (Fig. 1). Confirmed PTB means that positive results on fluorescent smear microscopy, liquid culture, or Xpert, whereas patients with NTM infection were precluded and assigned to the “non-TB” group. A total of 248 (57.14%) patients were bacteriologically confirmed with PTB on RTS examination, of which 200 and 48 were identified according to the sputum and BALF results, respectively.

**Fig 2:**
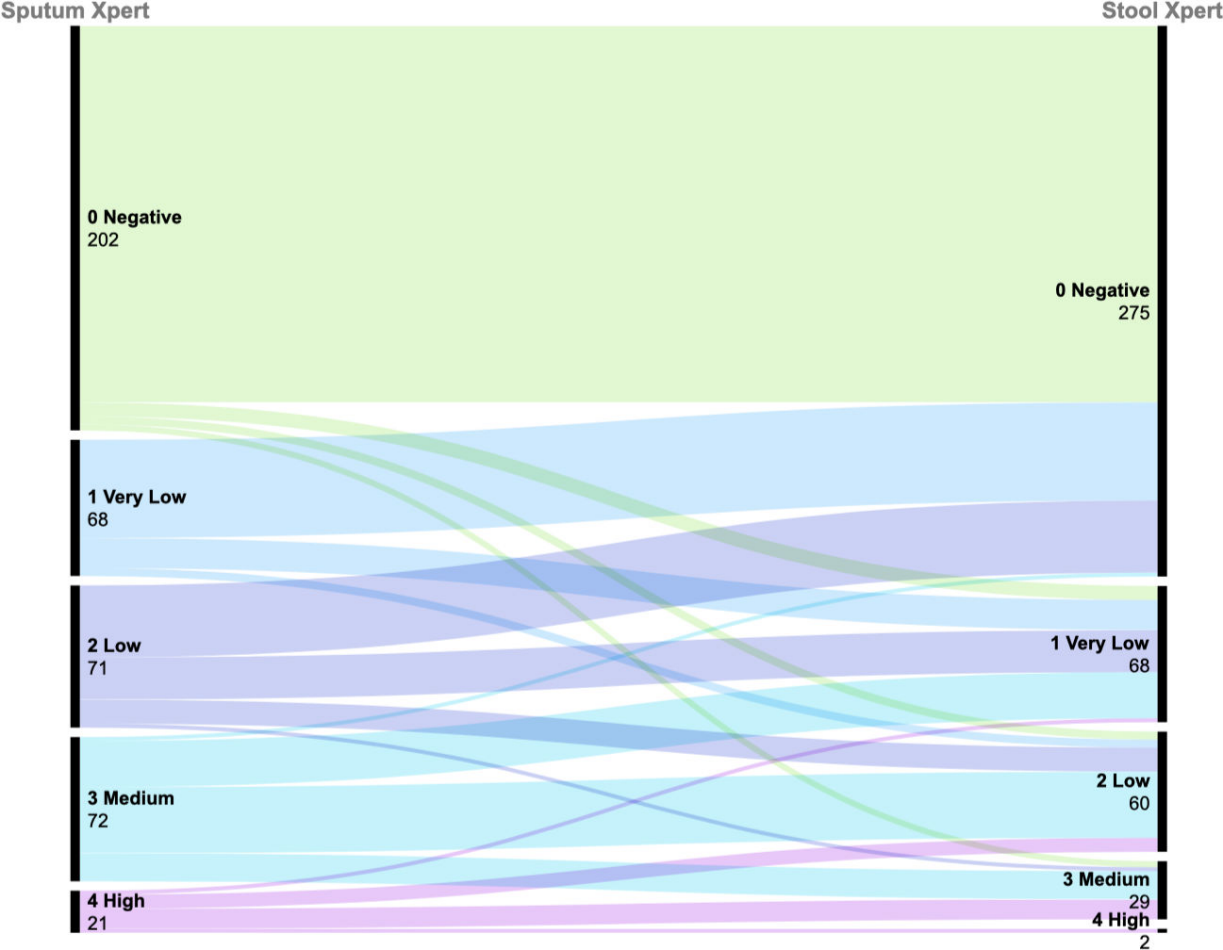
Alluvial plot of semiquantitative values of sputum Xpert and stool Xpert. Alluvial plot showing the relationship between semiquantitative measures of the cycle threshold recorded in stool and sputum by Xpert. Included are 434 participants who had undertaken sputum Xpert and stool Xpert.

Among the 351 patients with PTB, 232 (66.10%) and 159 (45.30%) were positive by Xpert on RTS and stool, respectively. Of the patients with negative Xpert results on RTS, 14 had stool specimens that were Xpert-positive (Fig. 2). Additionally, comparable sensitivities were obtained by Xpert on RTS (66.10% [95% CI: 60.85%–70.99%]) and Xpert on stool (45.30% [95% CI: 40.03%–50.67%]; *χ*^2^ = 30.764, *P* < 0.001). Lastly, Xpert on stool exhibited a specificity of 100.00% (95% CI: 94.49–100.00) (Table 2).

**TABLE 2:** Performance of different methods for pulmonary tuberculosis diagnosis on the different types of specimens using patient category as golden standard

Diagnostics	Specimen type	Confirm TB *n* = 248	Possible TB *n* = 103	Non-TB *n* = 83	Sensitivity % (95% CI）	Specificity % (95% CI）	PPV % (95% CI）	NPV % (95% CI）
Xpert	Stool	149/248	10/103	0/83	45.30 (40.03–50.67）	100.00 (94.49–100.00）	100.00 (97.06–100.00）	30.18 (24.89–36.04）
Xpert	RTS	232/248	0/103	0/83	66.10 (60.85–70.99）	100.00 (94.49–100.00）	100.00 (97.97–100.00）	41.09 (34.30–48.23）
Culture^*a*^	Stool	70/248	4/103	4/83	21.08 (17.01–25.80）	95.18 (87.45–98.44）	94.87 (86.69–98.34）	22.19 (18.06–26.94）
Culture^*a*^	RTS	190/248	5/103	9/83	55.56 (50.18–60.81）	89.16 (79.94–94.62）	95.59 (91.52–97.83）	32.17 (26.27–38.69）

^*a*^ Five patient with sputum or stool culture positive was identified to be infected by *M.intracellure* and classified to non-TB group.

**Fig 3:**
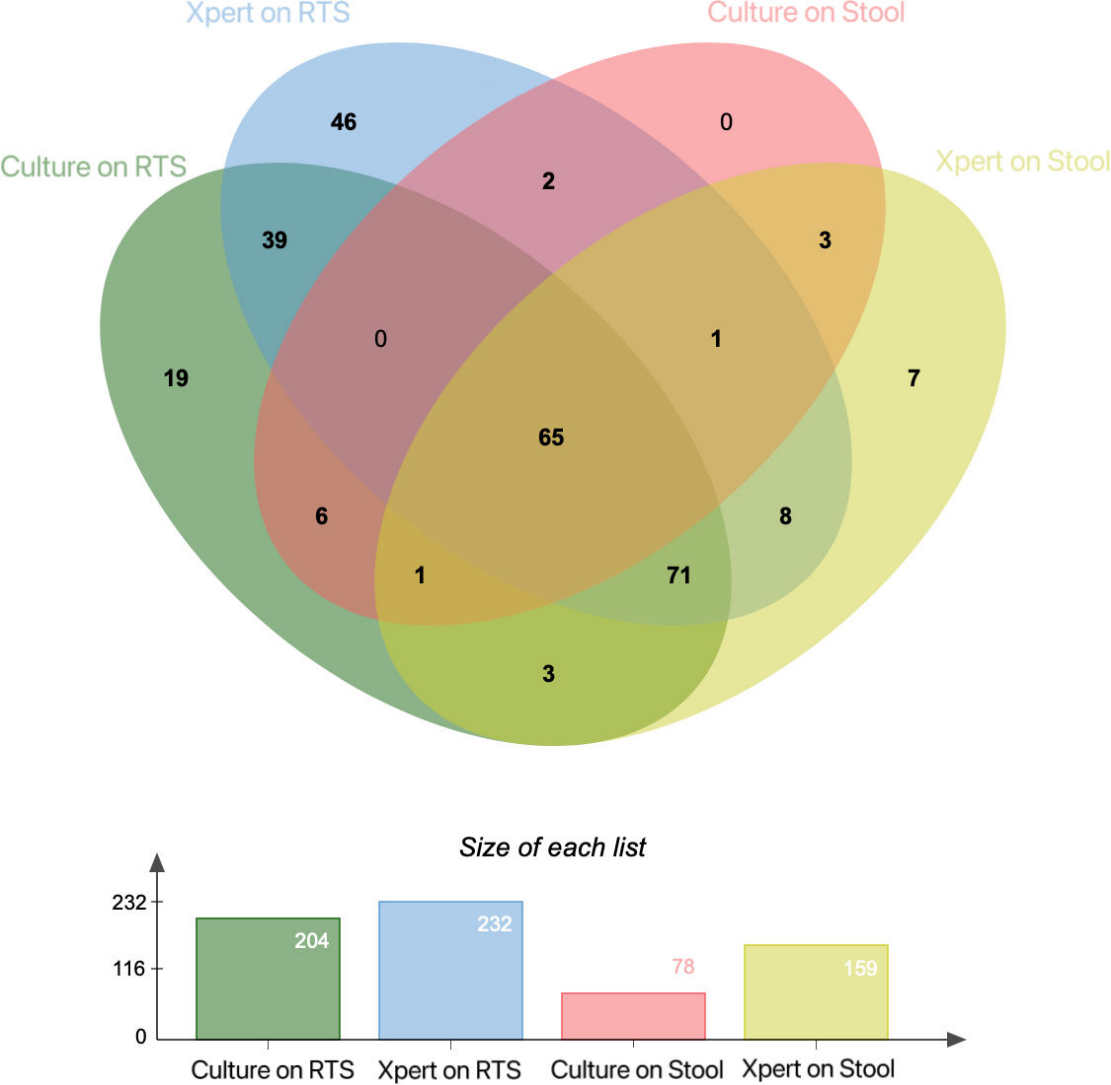
Venn diagram depicting bacteriological confirmation using culture, and Xpert MTB/RIF assays on RTS and stool specimens with presumptive pulmonary tuberculosis in adults.

Notably, 10 of the patients categorized with probable PTB according to the RTS results had positive results by Xpert on stool specimens. Therefore, additional testing with Xpert on stool proved valuable by showing positive TB results in 2.85% (10/351) of the patients with probable PTB. In contrast, 1.14% (4/351) of the patients with confirmed PTB were positive by culture on stool (Fig. 3). Furthermore, among all 48 patients with confirmed PTB on BALF examination, 16 were positive by Xpert on stool (CT Value, 29.2 ± 3.17) and seven were positive by culture on stool (CT Value, 29.6 ± 4.07). Using Xpert on RTS as the reference test, the sensitivity and specificity of Xpert on stool were 62.50% (95% CI: 55.90–68.68) and 93.07% (95% CI: 88.41–96.01), respectively (Table 3).

**TABLE 3:** Diagnostic accuracy of Xpert MTB/RIF assays on stool specimen compared with Xpert MTB/RIF assays on RTS specimen with presumptive pulmonary tuberculosis

		Xpert MTB/RIF on RTS^*a*^					
		Positive (*n* = 232）		Negative (*n* = 202）			
Diagnostics	Result	No.	%	No.	%	Sensitivity	Specificity
Xpert MTB/RIF on stool	Positive	145	62.50	14	6.93	62.50 (55.90 to 68.68)	93.07 (88.41 to 96.01)
	Negative	87	37.50	188	93.07		

^*a*^ RTS, respiratory tract specimens.

Among the 83 patients categorized in the non-TB group, five had positive cultures from the sputum and stool specimens, and the isolated strain was subsequently identified as *M. intracellulare*. Moreover, all Xpert results on sputum and stool were negative in this patient group. Thus, the specificity of Xpert on RTS and stool was 100% (83/83), while the specificity of MGIT 960 liquid culture on RTS and stool was 95.18% (79/83).

### Diagnostic yields using RTS and stool specimens

Of the 248 patients with bacteriologically confirmed PTB, Xpert on RTS had a diagnostic yield of 93.55% (232 patients), whereas Xpert on stool identified PTB in 149 (60.08%) patients (Table 4). Compared to Xpert, culture on stool demonstrated a lower diagnostic yield by detecting 74 patients among 351 diagnosed with TB. Of the 351 patients in the total diagnosed TB group, Xpert on stool specimens identified PTB in 159 (Table 4).

**TABLE 4:** Diagnostic yield of different laboratory assays on RTS and stool specimens among pulmonary tuberculosis

Investigations	No. of positive	Bacteriologically confirmed TB (%) (*n* = 248)	Total diagnosed TB^*a*^ (%) (*n* = 351)
Only smear on RTS	102	41.13 (102/248)	29.06 (102/351)
Only culture on RTS	195	76.61 (190/248)	55.56 (195/351)
Only Xpert on RTS	232	93.55 (232/248)	66.10 (232/351)
Culture plus Xpert on RTS	252	99.60 (247/248)	71.79(252/351)
Only smear on stool	45	17.34 (43/248)	12.82(45/351)
Only culture on stool	74	28.23 (70/248)	21.08(74/351)
Only Xpert on stool specimen	159	60.08 (149/248)	45.30(159/351)
Culture plus Xpert on stool	163	61.29 (152/248)	46.44(163/351)

^*a*^ Total diagnosed TB, bacteriologically confirmed TB plus probable TB cases.

## DISCUSSION

The diagnosis of TB in individuals with presumptive PTB who are unable to expectorate sputum is challenging, thus necessitating a non-invasive, non-sputum-based alternative for PTB diagnosis (23, 24). In recent years, stool specimens have been recommended as the initial specimen for TB diagnosis in children (25, 26). The utility of stool examination in patients with adult PTB has been previously evaluated in a few studies (17, 19, 27). For example, our previous study, which used patient categorization as the gold standard, showed that the sensitivity and specificity of Xpert on stool was 45.83% (95% CI: 35.73–56.28) and 100.00% (95% CI: 87.36–100.00), respectively (19). Compared to other specimen types, stool specimens have not been well assessed for their effectiveness in conventional testing and molecular diagnostics. Consequently, the usefulness of stool specimens in PTB diagnosis is not commonly recognized. In this study, we evaluated the performance of stool-based Xpert in diagnosing TB among adults in settings with high TB burden and low HIV prevalence in China. Although the sensitivity of Xpert on stool (45.3%) is somewhat lower than that of Xpert on RTS (66.1%), stool-based testing still offers significant advantages, particularly in situations where sputum or RTS samples are difficult or impractical to obtain. Despite the 20.8% difference in sensitivity, stool testing provides a non-invasive, easily accessible alternative, which is especially valuable in pediatric populations, immunocompromised patients, and resource-limited settings.

The WHO has recommended utilizing stool specimens for TB diagnosis in children; however, no standard operating procedure (SOP) for stool specimen processing is currently available (28). Two recent meta-analyses have revealed that the reported sensitivity of stool-based Xpert varies from 25% to 85%, with the differences in stool specimen processing methods being one of the reasons for this variation in sensitivity (16, 29). Our previous study employed a modified version of the stool processing method developed by de Haas et al. Moreover, the optimal procedure was suggested to involve a stool sample of 0.5 g, manual shaking, 30 min sedimentation, and processing using a 1:3.6 dilution ratio of the Xpert sample-processing reagent to stool sample (30). This centrifuge-free stool processing procedure was easy and resulted in high yields with a low invalid result and error rate of 0.77% (1/130). In the current study, we used our previous stool processing method to prepare the stool samples of 1 g, and only 13 of the 434 stool specimens had invalid results (3.0% [13/434]). Additionally, our method of stool processing was quick and simple and could avoid the loss of bacilli due to the absence of the centrifugation step. In light of the urgent need for an SOP for stool sample processing, our experience may provide valuable insights for establishing an SOP for stool specimen preparation for the Xpert assay.

Among the 83 patients in the non-TB group, four were culture-positive and Xpert-negative on sputum and stool examination. Among them, two patients were smear-positive on sputum and stool. Our recent study indicated that these test results were indicative of the presence of NTM, with the culture-positive and Xpert-negative findings having a sensitivity of 95.45% (105/110) for detecting NTM infection (31). Additionally, the isolates from the five patients with culture-positive and Xpert-negative results were identified as *M. intracellulare* by *16srRNA*, *rpoB*, and *hsp65* alignment. Thus, specimens with negative Xpert and positive smear/culture results may be an early reliable indicator for NTM infection.

Respiratory secretions are regularly discharged into the gastrointestinal tract (11), allowing the detection of MTB or its DNA in the stool of patients with PTB. Therefore, the relatively inferior performance of Xpert on stool compared to that of Xpert on RTS is expected. Generally, the incremental value of these extra tests on stool specimens was relatively weak. Only 11 additional patients with probable PTB exhibited bacteriological evidence on stool examination, resulting in a 3.13% increase in those with confirmed PTB. Moreover, given that the detection capacity of Xpert on stool (45.30% [159/351]) was inferior to that of Xpert assay on sputum (66.10% [232/351]), Xpert on stool may not be necessary for patients with enough sputum to maintain cost-effectiveness. Among the participants with negative Xpert results on RTS, 14 had Xpert-positive results on stool. Furthermore, 16 of the 48 patients with positive Xpert results on BALF were also Xpert-positive on stool specimens. Thus, we recommend that Xpert on stool specimens for diagnosing PTB in adults is a promising alternative strategy to the extremely invasive bronchoscope examination.

Our study has several limitations that should be considered. First, this study was a single-center investigation with a modest sample size. Hence, larger multicenter trials are required to expand the data supporting this strategy and account for the potential effects of patient variability on the overall performance of the tests. Second, the specificity and positive predictive value of Xpert on stool may be overestimated in this investigation because of the high TB burden in this study setting. Finally, no approved SOP is currently established for processing stool specimens. Consequently, significant variation may be introduced between studies using distinct stool processing techniques.

In conclusion, the diagnostic yield of Xpert on stool samples is lower than that of Xpert on RTS. Nevertheless, stool-based Xpert may be useful in detecting disease in patients with presumptive TB who cannot expectorate sputum, with stool serving as a good alternative specimen for sputum in such patients.

## MATERIALS AND METHODS

### Patient enrollment

From January 2021 to December 2021, patients with characteristic symptoms of TB, such as suggestive chest radiography, fever, weight loss, and cough lasting longer than 2 weeks, were recruited prospectively and consistently from the Beijing Chest Hospital. All eligible patients were ≥18 years.

### Patient categorization

Based on their clinical symptoms and specimens test results from smear, culture, and Xpert on RTS, the included patients were classified into one of the following three diagnostic categories:

1. Bacteriologically confirmed TB group: positive results using RTS on smear, culture, or Xpert, and determined to be in the presence of Mtb by species identification.
2. Probable TB group: negative results on smear, culture, or Xpert AND absence of other pulmonary abnormalities by medical imaging (including pneumonia, lung tumor, lung cyst, and interstitial lung diseases) AND favorable clinical response to anti-TB chemotherapy.
3. Non-TB group: other pulmonary conditions including NTM infection identified through pathological examination and laboratory tests. FM smear, Xpert, and MGIT results were all negative for MTB and other lung diseases identified through pathological examination and laboratory tests.

The gold standard for our investigation was patient categorization, where patients with bacteriological confirmation of TB and those with probable TB diagnosis were combined to form the total diagnosed TB group.

### Study procedures and specimen collection

This prospective cohort study assessed the performances of smear, culture, and Xpert in diagnosing PTB using stool samples from adults in the Beijing Chest Hospital from January 2021 to December 2021. Sputum was collected and underwent smear, culture, and Xpert tests. BALF (about 10 mL) was orally acquired as a substitute for sputum in patients who were unable to expectorate it. Concurrently, a stool sample was obtained from each enrolled patient and tested by Xpert, smear, and culture.

### Smear

According to the Mycobacteriology Laboratory Manual, smear microscopy of RTS (sputum or BALF) was performed with auramine-O staining (Baso, Guangzhou, Guangdong, China), and the acid-fast bacilli were detected by light-emitting diode microscopy (32).

### Culture tests and species identification

For the culture test, the fecal samples or RTS were treated with a final 1% *N*-acetyl-cysteine-NaOH for decontamination (20 min) and digested before inoculation into the growth vials for the MGIT 960 liquid culture (BD Diagnostic Systems, NJ, USA) based on the manufacturer’s instructions. For the culture positive strains, species identification was performed by sequencing of *16S rRNA*, *hsp65*, *rpoB*, and *16S-23S rRNA* internal transcribed spacer genes (33).

### Xpert assay on RTS

Following the manufacturer’s instructions, the Xpert assay was conducted using a 2:1 ratio of Xpert reagent to RTS. The cartridges were leveraged to the Xpert platform for automatic detection.

### Xpert assay on stool

The simple one-step (SOS) stool procedure (i.e., only including one release/sedimentation step) developed by de Haas et al. was the basis for the modified stool processing method used in this study (30). A stool sample of 0.8–1.0 g was collected into a 50 mL plastic tube, and 3 mL of the Xpert reagent was added to the sample in accordance with the manufacturer’s instructions. After the tube was shaken for 30 s, it was rested at room temperature for 5 min. Subsequently, the tube was again shaken vigorously for 30 s and rested for 10 min to allow the cell debris to settle via gravity sedimentation. Finally, 2 mL of the top liquid layer was transferred to the Xpert cartridge. Samples were generally examined on the same day of collection. In cases where the samples were not tested on the collection day, they were frozen at −80℃ until utilized.

### Data analysis

SPSS version 20 (IBM) was used to analyze the study data. The sociodemographic details, TB history, and symptom profiles of the included patients were compiled as proportions. The bacteriological confirmation results from culture and Xpert on RTS and stool samples were presented as a Venn diagram. An alluvial plot was constructed to depict the semiquantitative Xpert results of the sputum and stool samples. Sensitivity, specificity, positive predictive values, and negative predictive values for the culture and Xpert assay were calculated using the reference standard available at http://vassarstats.net.

The formulae were as following:

Sensitivity = True Positives/(False Negatives + True Positives)

Specificity = True Negatives/(False Positives + True Negatives)

Positive Predictive Value (PPV) = True Positives/ (False Positives + True Positives)

Negative Predictive Value(NPV) = True Negatives/(False Negatives + True Negatives).
